# In Vitro and In Vivo Anti-Aging Effect of Coffee Berry Nanoliposomes

**DOI:** 10.3390/molecules28196830

**Published:** 2023-09-27

**Authors:** Nisakorn Saewan, Ampa Jimtaisong, Nattakan Panyachariwat, Phanuphong Chaiwut

**Affiliations:** 1 School of Cosmetic Science, Mae Fah Luang University, 333, Moo.1, Thasud, Muang, Chiang Rai 57100, Thailand; ampa@mfu.ac.th (A.J.); nattakan.pan@mfu.ac.th (N.P.); phanuphong@mfu.ac.th (P.C.); 2 Cosmetic and Beauty Innovations for Sustainable Development (CBIS) Research Group, Mae Fah Luang University, 333, Moo.1, Thasud, Muang, Chiang Rai 57100, Thailand; 3 Green Cosmetic Technology Research Group, Mae Fah Luang University, 333, Moo.1, Thasud, Muang, Chiang Rai 57100, Thailand

**Keywords:** anti-aging, antioxidant, coffee berry, elasticity, liposomes, nanoliposome, melanin, skin color

## Abstract

Encapsulation of bioactive compounds in the liposome system provides several advantages, such as enhancing the stability and lowering the toxicity of active compounds. Coffee berry extract (CBE) has previously been established to have in vitro anti-aging properties and to retard the aging of human skin. The purposes of this study were to encapsulate CBE in nanoliposomes and to assess its stability and in vitro anti-aging potential in human dermal fibroblasts (HDF), as well as in healthy human skin. In the HDF model, anti-aging potential was determined by nitric oxide (NO) and collagenase inhibition assays and a superoxide dismutase (SOD) activity assay, whereas in healthy human skin (in vivo), the skin elasticity and brightness were examined. First, liposomal CBE (L-CBE) was created with a particle size of 117.33 ± 2.91 nm, a polydispersity index (PDI) of 0.36 ± 0.03, and a zeta potential of −56.13 ± 1.87 mV. The percentages of encapsulation efficacy (%EE) and loading efficacy (%LE) were 71.26 ± 3.12% and 2.18 ± 0.18%, respectively. After undergoing a 12-week stability test, the L-CBE retained more phenolic content than the free CBE when stored at 4 °C, room temperature, and 45 °C. Compared to free CBE, the L-CBE demonstrated a more consistent, elevated, and prolonged release of phenolics from the lipid system. In human dermal fibroblasts, L-CBE showed lower toxicity, and at its maximum nontoxic concentration (10 mg/mL), it exhibited slightly higher anti-aging effects than CBE, including NO inhibition, enhanced SOD activity, and anti-collagenase activities. In clinical trials (30 volunteer subjects), none of the participants’ skin was irritated when the L-CBE, the CBE, or base creams were applied. After 2 weeks of application, the L-CBE and CBE creams both demonstrated an improvement in skin elasticity and a reduction in melanin levels, and after 4 weeks, L-CBE cream showed a significantly greater improvement in skin elasticity and lightening. The results demonstrate that the encapsulation of the CBE in liposomal systems could increase its stability and skin penetration, reduce its toxicity, and maintain its anti-aging effect, which is powerful enough to be exploited in anti-aging and whitening agents for application in cosmetics and cosmeceuticals.

## 1. Introduction

Aging is a complex biological phenomenon characterized by a gradual and ongoing decline in cellular and organismal functionality over the course of a lifetime. This process ultimately leads to senescence, which is influenced by both extrinsic and intrinsic aging. Signs of aging encompass several manifestations, such as hyperpigmentation, diminished skin elasticity and laxity, the presence of fine lines and wrinkles, telangiectasia, uneven skin texture, enlarged pores, periorbital puffiness, keratosis, and additional related indicators [1]. Several research investigations have been conducted globally in the search for active substances that possess the capacity to inhibit and decelerate the manifestations of aging on the skin. Plant extracts are widely utilized in anti-aging cosmetics due to their antioxidant, immunostimulant, radical scavenging, and UV protection qualities, in addition to other functions [1,2].

The coffee berry, which is the fruit of the coffee plant, exhibits a spherical shape and displays colors of crimson and purple when fully ripe. Each individual coffee berry often consists of a pair of seeds, referred to as green beans or green coffee beans due to their characteristic green coloration [3]. The coffee husk and pulp are discarded as waste during the coffee production process, prior to the subsequent roasting phase that imparts the characteristic aroma of coffee, a widely consumed beverage worldwide. Based on the existing literature, it has been established that certain components of the coffee berry fruit have anti-aging properties. The entire fruit exhibits considerable potential for use in formulations aimed at counteracting the effects of aging. In a recent investigation, we found that CBE had a high phenolic content and good antioxidant activity in terms of ferric reducing antioxidant power, 1,1-diphenyl-2-picrylhydrazyl radical (DPPH) radical scavenging, NO and collagenase inhibition, and enhanced SOD activities [4,5]. McDaniel found that using 1% and 0.1% CBE in cream and cleanser, respectively, twice a day improved the skin’s appearance, fine lines and wrinkles, roughness and dryness, and pigmentation. The application of the items reduced collagenase (MMP-1) and IL-1 (inflammatory mediators in skin biopsies), and also increased collagen type I and collagen type IV [6]. Caffeine and chlorogenic acid, among other antioxidant and prooxidant chemicals, are abundant in coffee [7]. Caffeine is an antioxidant that suppresses lipid peroxidation, protects cells from free radical damage, enhances cell oxygenation and microcirculation, and accelerates cell metabolism [8,9]. It is mostly used in hair care cosmetics to prevent hair loss and stimulate new and healthy hair growth by inhibiting the 5-alpha-reductase enzyme [10,11,12]. It also provides skin anti-aging properties, including enhanced keratinocyte cell proliferation and inhibited collagenase activity [4]. Chlorogenic acid is an active polyphenol with anti-inflammatory, antioxidant, and antimicrobial effects [13,14]. It has antioxidant activity which promotes collagen and elastin fiber in skin fibroblasts, providing anti-inflammatory and wound-healing effects that are linked to their ability to slow the signs of aging on the skin [15,16,17,18].

Liposomes are drug delivery systems in the form of spherical vesicles which consist of a phospholipid bilayer membrane. Liposomes provide several advantages for application, such as enhancing the stability of active compounds, having good biocompatibility, and lowering toxicity in the encapsulation system [19]. Moreover, liposomes have been reported as penetration enhancers for dermal and transdermal delivery, resulting in an increased level of efficacy and therapeutic index [20]. The encapsulated molecules in liposomes are better absorbed in the skin and penetrate the stratum corneum through intercellular spaces [21]. Liposomes can be created using natural phospholipids and derived phospholipids with mixed lipid chains. Egg and soybean phosphatidylcholine are most commonly used. Due to being water-insoluble, phosphotidylcholine molecules create bilayer-shaped planner sheets that lessen the unfavorable reaction between long fatty chains of hydrocarbon and the excessive water phase [22]. Other essential elements of more stable liposomes are steroids, such as cholesterol or its derivatives. The primary steroid typically employed in liposome synthesis is cholesterol, which has the effect of decreasing membranous permeability towards hydrophilic molecules, enhancing the fluidity of the phospholipid bilayer, and increasing the stability of the bilayer in the biological environment [23,24]. Liposomes of green coffee and medium-roasted coffee extracts have been separately prepared and have strong antioxidant properties [25]. In another study, spent coffee grounds maintained their antioxidant activity through the supercritical assisted liposome production method [26].

To the best of our knowledge, research on coffee liposomes has primarily concentrated only on in vitro antioxidant tests; however, the stability, skin penetration, and anti-aging benefits of coffee liposomes have not been extensively discussed in the literature. This research aims to investigate the stability, skin penetration, and in vitro anti-aging effects of L-CBE encapsulation in HDF cells and the in vivo anti-aging effect of L-CBE cream formulation in human skin. The stability of L-CBE, based on its total phenolic content, was also examined under different storage temperatures—4 °C, room temperature, and 45 °C—for 12 weeks. The anti-aging properties of the liposomes in HDF cells were investigated using assays for collagenase inhibition, superoxide dismutase activity, and nitric oxide (NO) inhibition. The anti-aging effectiveness of L-CBE in cosmetic formulation was clinically evaluated by measuring skin color (melanin content), firmness (gross elasticity (R2), net elasticity (R5), and immediate recovery elasticity (R7)).

## 2. Results

### 2.1. Determination of Chlorogenic Acid and Caffeine

**Figure 1:**
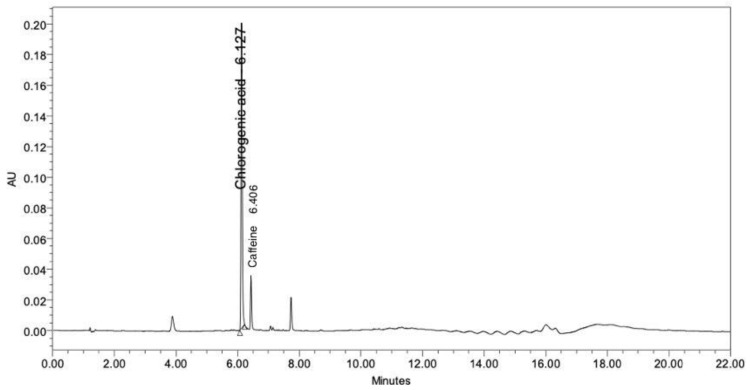
Chromatogram of the CBE by HPLC.

Coffee is rich in biologically active alkaloids and phenolics. The well-known major bioactive constituents of coffee are caffeine and chlorogenic acid [27,28]. Therefore, in this study, the CBE was analyzed to assess the levels of chlorogenic acid and caffeine using HPLC. The chromatogram of the CBE at a UV detection wavelength of 280 nm showed two major peaks, which were identified as chlorogenic acid and caffeine at retention times of 6.127 and 6.406 min, respectively (Figure 1). The concentrations of the chlorogenic acid and the caffeine were calculated from the peak area against the standard calibration curve and showed values of 131.85 and 6.44 µg/mL, respectively.

### 2.2. Preparation and Characterization of the Liposomal CBE

**Figure 2:**
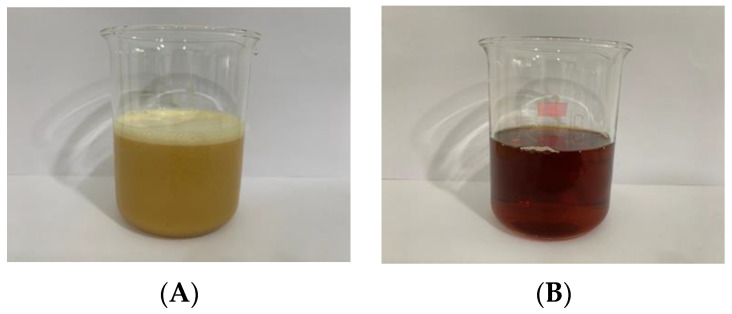
Appearance of L-CBE before (**A**) and after (**B**) size reduction.

In this investigation, phosphatidylcholine and cholesterol were used as the constituents for liposome formation. Liposomes were first prepared via the film method, with a ratio of phosphotidylcholine and cholesterol of 15:1, and a light brown translucent liquid was obtained. After 5% of the CBE was encapsulated into the liposome system, the L-CBE was derived as a darker liquid. Over the course of five cycles in a microfluidizer operating at 25,000 psi, the liposomes transformed from a translucent to a transparent liquid as a result of the reduction in particle size (Figure 2).

#### 2.2.1. Particle Size and Zeta Potential Measurement

The particle size, PDI, and zeta potential of the L-CBE was investigated by Zetasizer. Before size reduction, the particle size of the liposome was 675.26 ± 24.8 nm, which was noticeably smaller (117.33 ± 2.91 nm, monodisperse) after size reduction. A similar result was found in the preparation of the liposomes using a microfluidizer at pressures of 15,000, 15,000, and 20,000 psi for three cycles. The results indicated a decrease in particle size from an initial value of 2570 ± 2120 to 194 ± 18, 189 ± 4, and 154 ± 22 nm, respectively [29]. A study by Carugo et al. also reported that the microfluid method (1 cycle) creates reproducible liposomes containing cationic dimethyl dioctadecyl ammonium bromide with an average size of 90–150 nm [30].

The polydispersity index (PDI) is an important factor that describes the width or spread of the particle size distribution, which ranges from 0.0–1.0. A greater PDI value will result in a wider variety of particle sizes. According to Lacatusu (2012), the appropriate dispersion value must be close to 0.0 or less than 0.5 [31]. The PDI value of the prepared L-CBE was 0.36 ± 0.03, which should provide good stability by preventing coagulation and phase separation.

Zeta potential, which measures the strength of the repulsion or attraction between particles, is a key technique for assessing the physical stability of the nanocolloid system. Large positive or negative values of a nanoparticle’s zeta potential cause electrostatic repulsion between individual particles and lead to good physical stability of nanosuspensions. Normally, zeta potentials greater than +30 mV or less than −30 mV are regarded as stable [32]. The L-CBE showed a zeta potential of −56.13 ± 1.87 mV, which represented repulsion between particles providing good stability against sediment forming over time.

#### 2.2.2. Encapsulation Efficiency and Loading Efficacy

Encapsulation efficiency (EE) is the proportion of active compounds that are successfully encapsulated in nanoparticles. It refers to the ability of the wall material to enclose the core material, which depends on several variables, including the chemical nature of the core (molecular weight, chemical functionality, polarity, and volatility), the properties of the shell material, and the technique and conditions used for preparation [33,34]. The results showed that the L-CBE yielded an EE of 71.26 ± 3.12%, slightly higher than the loading of green coffee bean extract in the liposome/polyhydroxybutyrate, which obtained 67.50% EE [35]. Loading efficiency (LE), which refers to the percentage of the bioactive ingredient present in the nanoparticle system (L-CBE), was found to be 2.18 ± 0.18%.

#### 2.2.3. Stability Test

**Figure 3:**
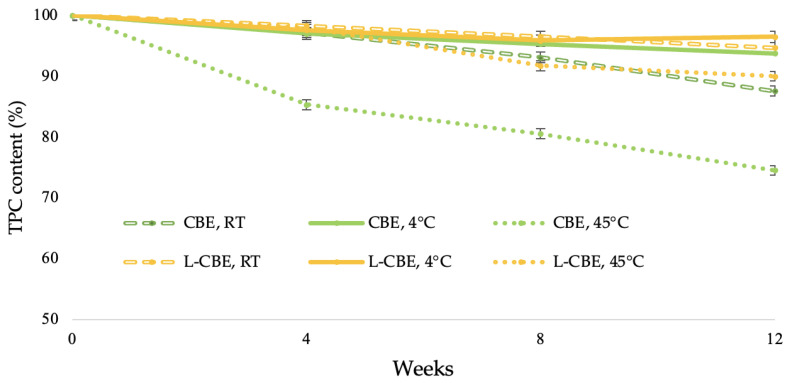
Stability of the CBE and the L-CBE at various storage conditions.

The stability of natural extracts in cosmetic and pharmaceutical fields is a concern due to the phytochemicals being easily decomposed, thus causing an unfavorable impact on the quality of the extract [36]. The exposure to high temperatures plays an important role in terms of polyphenol stability, particularly phenolic compounds such as chlorogenic acid, caffeic acid, and ferulic acid. Under the dual factors of light and temperature, these phenolic compounds readily decompose [37,38]. To increase the stability of active compounds under stress conditions, liposome encapsulation is one of the most widely used methods for maintenance. Thus, to evaluate the effects of various temperatures on the accelerated stability of the CBE and the L-CBE, the samples were stored at 4 °C, RT, and 45 °C for 12 weeks. After 4 weeks, the L-CBE retained phenolic contents of 98.27, 97.53, and 97.98% at 4 °C, RT, and 45 °C in storage conditions, which were higher than those of the free CBE, i.e., 97.13, 96.96, and 85.30%, respectively. At the end of the experiment (week 12), the L-CBE retained phenolic contents of 94.66, 96.45, and 89.97%, while the CBE decreased to 93.72, 87.55, and 74.49% at 4 °C, RT, and 45 °C, as displayed in Figure 3. At the low temperature of 4 °C and at ambient temperature, the retained phenolics of both samples were higher than 90%. However, at a high temperature, the retained phenolics in the free CBE quickly decreased to 75%, while that of the encapsulated CBE was about 90%. The results showed that a high temperature exhibited the greatest effect on the stability of the samples. Due to the high temperature’s degradation of the structure of the polyphenols in extract [39,40], they could be affected by their oxidation [41,42]. The evidence showed that encapsulation in liposomes could prevent the degradation of the extract under high thermal conditions. The results, also corresponding to Shariare’s work on liposome preparation by using egg phospholipids, provided significant better bioavailability and stability when compared to a single extract [43].

#### 2.2.4. In Vitro Permeation Measurement

**Figure 4:**
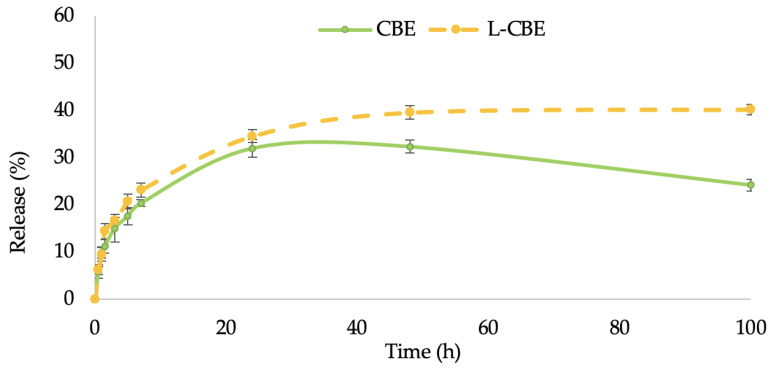
In vitro permeation of total phenolic content of the free CBE compared to the L-CBE.

In this study, the permeation ability through the stratum corneum of the CBE-loaded liposomes in comparison to the unloaded CBE was performed by investigating the controlled release of samples through cellulose acetate membranes with a 0.45 µm pore size, which were used as barrier and release media. The release of samples from liposomes followed by permeation through the membrane to the receptor fluid was evaluated using the Franz diffusion cell. The release (%) of phenolic content from the free CBE and the CBE loading in liposomes were plotted versus time. The release rates of both samples increased quickly for the first 6 h before slowing down. Thereafter, the release rates of the CBE encapsulation in the liposomes increased gradually and steadily over the period of time, but the rate of release from the free CBE increased progressively during the first 24 h and decreased over a period of 48 to 100 h (Figure 4). Accordingly, the skin penetration of liposomes is also influenced by their size. Liposomes smaller than 300 nm can permeate deeper skin layers, while liposomes smaller than 70 nm have been found to be the most efficient size for dermal distribution. Liposomes larger than 600 nm remained on the epidermis rather than penetrating deeper layers of the skin [44]. The greater skin penetration of the L-CBE encapsulation is caused by its small particle size (117.33 nm), allowing for basic skin absorption.

### 2.3. Cytotoxicity

**Figure 5:**
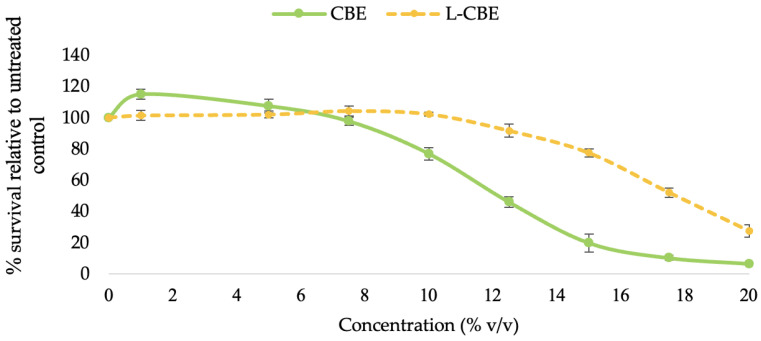
Cytotoxicity of various concentrations of the CBE and L-CBE on HDF cells.

The cytotoxicity of the free CBE and the L-CBE was first assessed by subjecting samples of a range of concentration (0–20 mg/mL) to human dermal fibroblast (HDF) cells. The pattern of cytotoxicity was the same in all test samples and was dose-dependent. The CBE (≤5.0 mg/mL) and the L-CBE (≤10.0 mg/mL) showed non-cytotoxicity to HDF cells at the low dose and also boosted cell proliferation by a small amount. The viability of the cells was reduced, as expected, as the sample concentration increased (Figure 5). The CBE demonstrated 76.84% cell vitality at a dosage of 10.0 mg/mL, whereas the L-CBE had a greater cell viability of 102.19%. At higher doses (12.50 mg/mL), the L-CBE also demonstrated a higher cell vitality of 91.62%, while the CBE exhibited a low cell viability of only 45.91%. Based on dose–response data compared to the control (untreated cells), the test sample concentration would result in 50% (IC_50_) of the cells surviving was determined. The L-CBE had a considerably higher IC_50_ (17.66 ± 0.35 mg/mL) compared to the free extract (12.92 ± 0.36 mg/mL), indicating a lower level of toxicity. Therefore, the test concentrations in further experiments were set at the highest non-toxic doses of the CBE (5 mg/mL) and the L-CBE (10 mg/mL).

### 2.4. Anti-Aging Activities

The anti-aging efficiency levels of the samples were evaluated using three different methods: SOD activity and NO and collagenase inhibition activities. In prior research, chlorogenic acid and CBE showed potent activities in all assays, and caffeine strongly inhibited only collagenase [4,45].

#### 2.4.1. Antioxidant Activity

**Figure 6:**
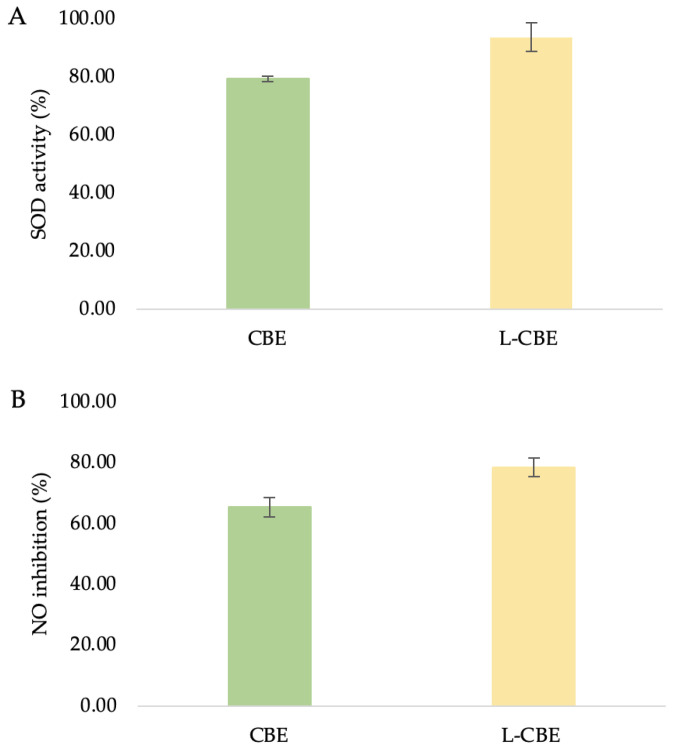

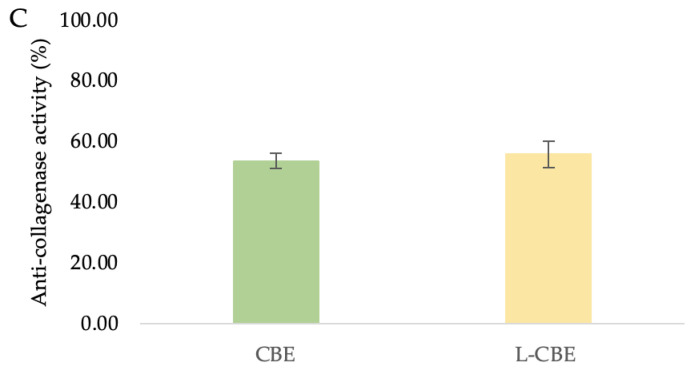
The superoxide dismutase (**A**), nitric oxide inhibition (**B**), and anti-collagenase (**C**) activities of the CBE and L-CBE on HDF cells.

SOD, the main antioxidant enzyme, catalyzes the interaction between oxygen (O_2_) and hydrogen peroxide (H_2_O_2_) to shield cells from the harmful effects of superoxide radicals (O_2_) [46]. Oxidative stress is brought on by an imbalance between the quantities of antioxidants and free radicals, which was seen in the decreased activity of SOD antioxidant enzymes [47]. SOD is a significant antioxidant found in human fibroblasts which contributes to the effect of oxidative stress on telomere shortening [48]. Additionally, encouraging SOD activity can help to minimize oxidative stress and photo-induced skin aging. To assess and examine the samples’ capacity to increase SOD antioxidant activity, a SOD determination kit that enables a practical assay by creating a water-soluble formazan dye upon reduction with a superoxide anion was utilized. Since SOD inhibits xanthine oxidase activity, decreased O_2_ is linearly related to that activity. As a result, the reduction in the color development of WST-1 formazan can be used to calculate the level of SOD activity. At the highest non-toxic doses of the tested samples, in comparison to the extract treatment (79.27 ± 0.96%), the liposomes’ treatment resulted in significant higher SOD activity (93.58 ± 9.45%) (Figure 6A).

#### 2.4.2. NO Inhibition Activities

Under normal physiological circumstances, NO functions as an anti-inflammatory agent and is involved in a variety of inflammatory disorders. However, NO overproduction is regarded as a pro-inflammatory mediator that causes inflammation when conditions are abnormal [49]. Moreover, NO is also a potent immunological mediator with strong cytotoxic and wound-healing effects [50]. Therefore, a reliable method by which to identify cell inflammation is to measure NO levels. In this work, HDF was treated with test samples and the amount of NO generation in the culture medium was measured using Griess reagent. With the same trend as SOD activity, the L-CBE showed a slightly higher level of NO inhibition at 78.50 ± 3.01% in comparison to the extract (65.30 ± 3.14%) (Figure 6B). The cause of this result could be the controlled release and penetration of active ingredients from the lipid bilayer in the encapsulation system.

#### 2.4.3. Anti-Collagenase Activity

The primary causes of the clinical signs of aging skin, such as wrinkles, sagging, and laxity, indicate the degradation of interstitial collagens and changes in the extracellular matrix [51,52]. Collagenase inhibition has been suggested as a method by which to limit the advancement of drooping as well as the loss of skin elasticity [53]. Therefore, the investigation of collagenase inhibition ability is the most frequently used assay to evaluate the anti-aging benefits of various natural substances to be utilized as cosmeceutical components. In our previous study, the CBE showed the highest significant inhibition of MMP-1 at 65.88%, which was higher than its major compounds, i.e., chlorogenic acid (53.19%) and caffeine (43.47%) [45]. To investigate the collagenase inhibition activity of the CBE loading in liposomes, bioassays were performed with a member of the group of matrix metalloproteinases, MMP-1. Inhibition of collagenase by the free CBE was slightly lower (53.72 ± 2.46%) than that of the L-CBE (55.96 ± 4.45%) (Figure 6C).

### 2.5. Formulation

**Figure 7:**
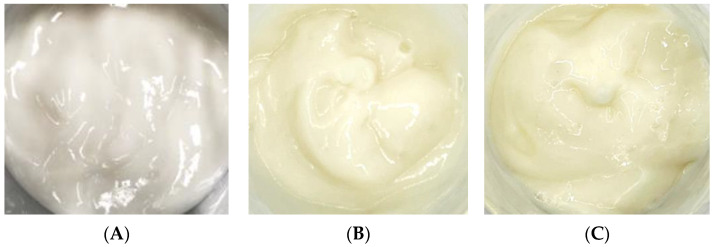
Emulsion cream base (**A**) with the CBE (**B**) and the L-CBE (**C**).

After adding the free CBE and liposome encapsulation of the CBE to an emulsion cream base as anti-aging components, both products exhibited light yellow colors, whereas the cream base (B cream) appeared as a white cream (Figure 7). The cosmetic formulation had pH values of 5.06 and 5.14 for the CBE and the liposomal CBE creams, respectively. In general, the texture, spreadability, thickness, and smoothness of both creams were noticeable. Overall, both creams were satisfactorily acceptable for topical application, with appreciable appearance, color, and texture; easy absorption; good spreadability; and non-greasiness.

### 2.6. Clinical Study

#### 2.6.1. Skin Irritation Testing

The skin irritation patch test was performed on 30 volunteer subjects. The erythema and edema of all tested samples were evaluated after 30 min and after 24 h patch removal. Slight erythema on the subjects’ skin was observed where sodium lauryl sulfate had been applied, with an M.I.I value of 0.13 after the 30 min patch had been removed. However, after 24 h, it had stopped showing erythema. All tested creams and deionized water indicated that no erythema occurred with an M.I.I. of 0.00. In addition, there was no edema in any of the tested samples (M.I.I. was 0.00). The outcome was consistent with various test reports. The application of hair tonic with 10% CBE on the scalp did not irritate human skin [10]. Coffee silver skin extract demonstrated no irritancy in an in vitro test on human epidermis and in an in vivo test on subjects’ skin when patched for 48 h [54]. Amnuaikit et al. reported that the gel base formula containing 3% caffeine caused no signs of discomfort after being applied on subjects’ arms for 24 h [55]. Both coffee extract and the major compounds in coffee have also been reported for their nonirritating properties. These results revealed that creams containing coffee extract can be considered safe and did not irritate human skin.

#### 2.6.2. Efficacy Test

Caffeine is one of the most common alkaloids found in coffee, and can be absorbed into both rat and human skin membranes (in vitro test) with small amounts of caffeine remaining [56,57]. The results of Meesen [58] showed that caffeine in O/W emulsion can penetrate through pig skin (in vitro test). Transepidermal water loss (TEWL) is the amount of water lost from the stratum corneum per skin area unit, which reflects the integrity of the skin barrier function and has previously been used to indicate healthy or unhealthy (damaged) human skin. High rates of TEWL indicated a weak skin barrier function, which can alter skin dryness and accelerate aging. In contrast, lower TEWL helps to restore the skin’s barrier and to preserve the skin’s moisture [59,60]. Caffeine (0.5%) in gel base has been effective in increasing the skin barrier function, and reduced transepidermal water loss (TEWL) in subjects’ skin [61]. Additionally, an in vivo investigation on mouse skin tissue showed that caffeine can shield the skin from oxidative-stress-related skin senescence [62]. Chlorogenic acid is a well-known free radical scavenging antioxidant [13]. It has shown anti-aging properties through decreasing reactive oxygen species (ROS) levels, the primary cause of aging, thus inhibiting related skin-aging enzymes such as MMP-1 and elastase and increasing hyaluronic acid and collagen contents in fibroblast cells [17,18,35,45,63]. Therefore, coffee extract, caffeine, and chlorogenic acid have the potential to prevent premature skin aging.

**Figure 8:**
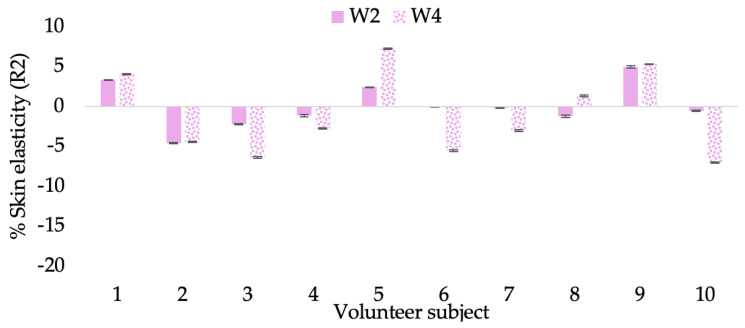
Changing percentage of elasticity (R2) of each volunteer subject after application of B cream.

**Figure 9:**
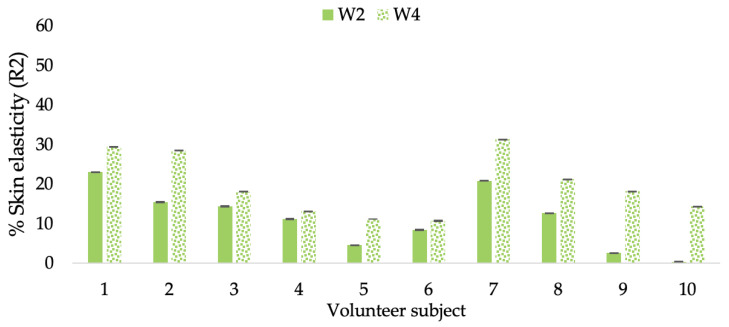
Changing percentage of elasticity (R2) of each volunteer subject after application of CBE cream.

**Figure 10:**
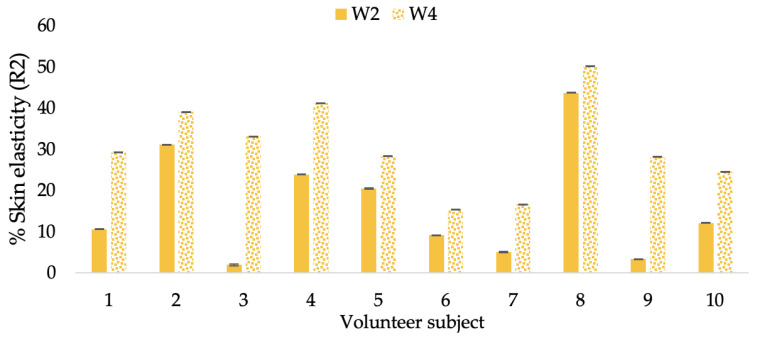
Changing percentage of elasticity (R2) of each volunteer subject after application of the L-CBE cream.

**Figure 11:**
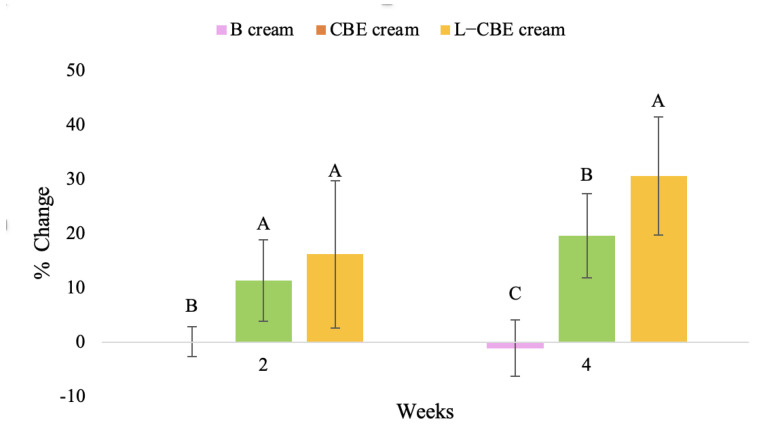
Changing percentage of elasticity (R2) of all volunteer subject subjects after product application. Data with different letters (A, B, and C) indicate significant differences (*p* < 0.05) between creams.

**Figure 12:**
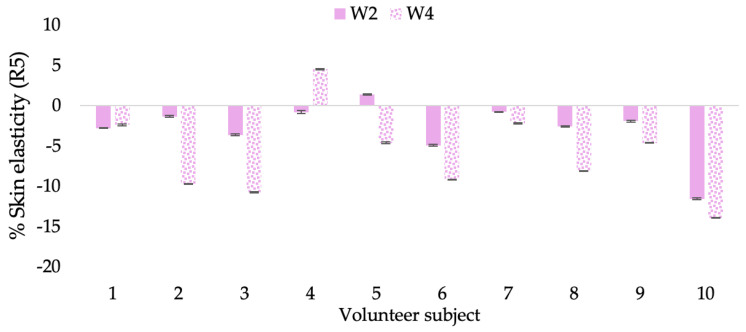
Changing percentage of elasticity (R5) of each volunteer subject after application of B cream.

**Figure 13:**
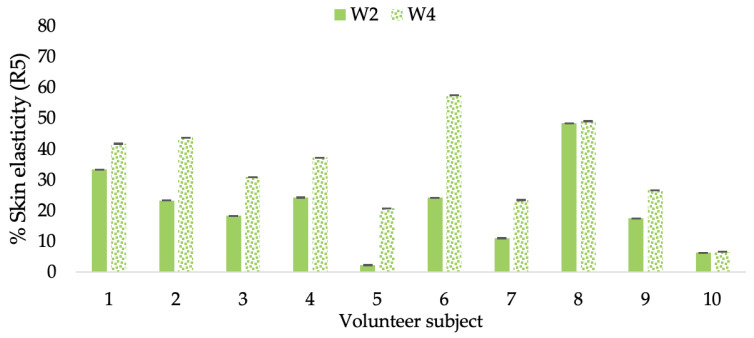
Changing percentage of elasticity (R5) of each volunteer subject after application of CBE cream.

**Figure 14:**
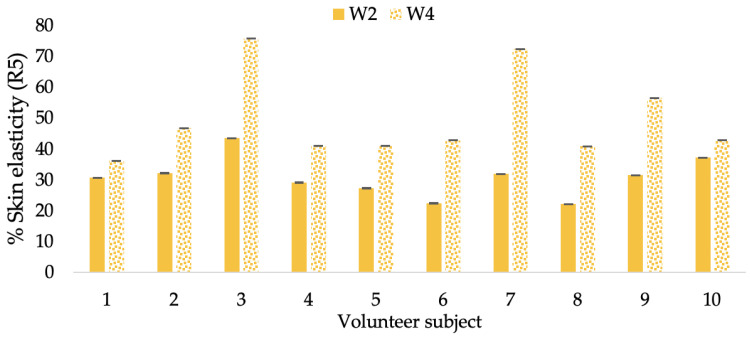
Changing percentage of elasticity (R5) of each volunteer subject after application of L-CBE cream.

**Figure 15:**
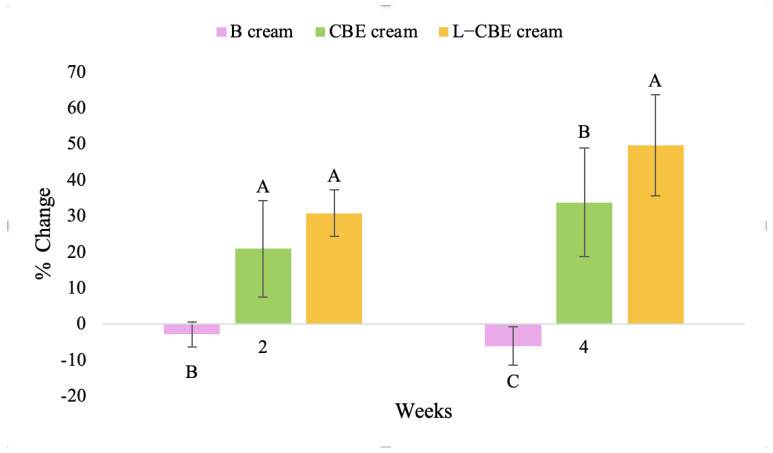
Changing percentage of elasticity (R5) of all volunteer subject subjects after product application. Data with different letters (A, B, and C) indicate significant differences (*p* < 0.05) between creams.

**Figure 16:**
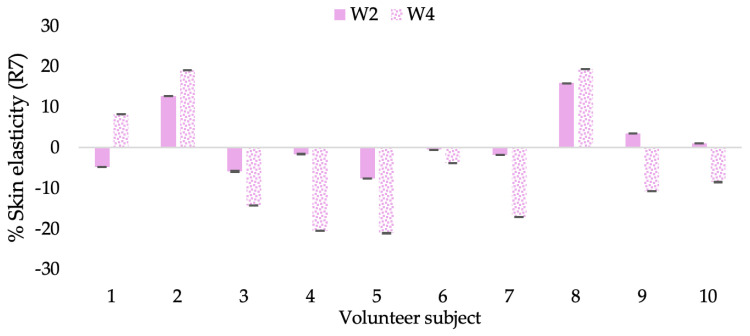
Changing percentage of elasticity (R7) of each volunteer subject after application of B cream.

**Figure 17:**
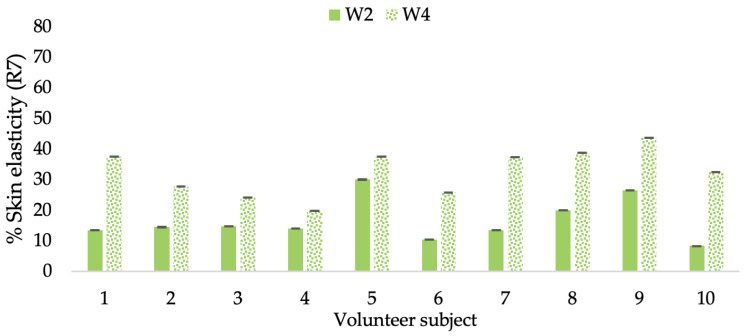
Changing percentage of elasticity (R7) of each volunteer subject after application of CBE cream.

**Figure 18:**
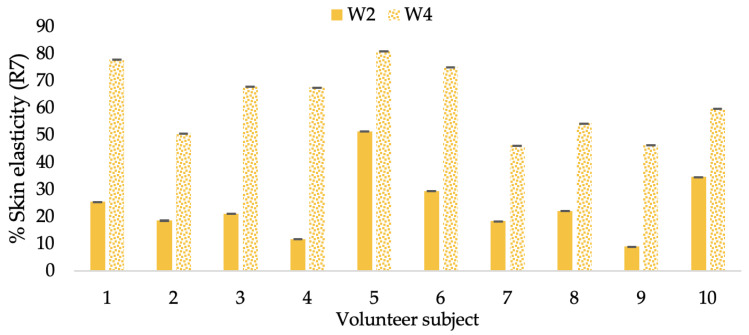
Changing percentage of elasticity (R7) of each volunteer subject after application of L-CBE cream.

**Figure 19:**
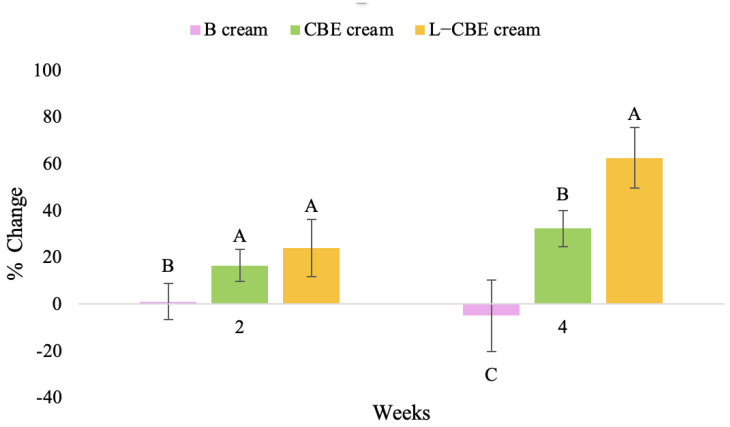
Changing percentage of elasticity (R7) in all volunteer subjects after product application. Data with different letters (A, B, and C) indicate significant differences (*p* < 0.05) between creams.

A typical element of aging is the loss of skin elasticity, which causes wrinkles and fine lines as people age. Accordingly, skin elasticity is a significant indicator of skin aging and is a measure to assess skin aging. A cutometer is a device for measuring the mechanical and viscoelastic characteristics of skin by measuring the response of skin to an applied suction force [64,65]. A cutometer was employed in this study to evaluate the anti-aging effectiveness of the L-CBE. Once the R parameters (R2, R5, and R7) had been determined, all values could be reported as elasticity. R2 refers to the skin’s overall elasticity and how resistant it is to mechanical force compared to its reversal. R5 is the net elastic, which is an elastic portion of the suction part versus an elastic portion of the relaxation part. R7 represents the ratio of immediate recovery to total deformation, known as the biological elasticity. The amount of elasticity is in relation to the final distension. If the skin were more elastic, the R values would have been closer to 1.0000 (100%). Thirty volunteer subjects with an average age of 42 ± 11.7 years participated in this single blind test. The oldest subject was 60 years old, while the youngest was 24. The participants were randomly divided into three groups of ten subjects. The first group applied a placebo cream without any active ingredient (B cream), while the second and third groups applied a cream containing 5% of the free CBE and the loaded liposomes (CBE and L-CBE creams, respectively). The participants were instructed to apply the test creams on their facial skin for 4 weeks. Following application, the elasticity of the skin was measured in triplicate at week 0 (baseline), week 2, and week 4. The variations in each subject’s skin elasticity throughout the 4-week product therapy experiment and the percentage changes in the skin elasticity (R2, R5, and R7) of each group were calculated as displayed in Figure 8, Figure 9, Figure 10, Figure 11, Figure 12, Figure 13, Figure 14, Figure 15, Figure 16, Figure 17, Figure 18 and Figure 19.

The results after using the cream for two weeks revealed that volunteer subjects who used the CBE and L-CBE creams experienced greater changes in skin elasticity (R2, R5, and R7) than those who used the placebo cream (*p* < 0.05). The change in skin gloss elasticity (R2) was significantly enhanced as compared to the baseline at 11.32 and 16.14% and continuously increased from the starting point, with 19.52 and 30.54% for the CBE and the L-CBE creams, respectively, after being applied for 4 weeks (Figure 8, Figure 9, Figure 10 and Figure 11). The net skin elasticity (R5) continuously increased the course of the investigation, reaching its highest levels at the end of the study, with maximum values of 33.75 and 49.63% for the CBE and L-CBE creams, respectively, in comparison to the baseline (Figure 12, Figure 13, Figure 14 and Figure 15). The biological elasticity (R7) increased noticeably in the second week of the trial, with 18.49 and 24.07%, and appreciably in the fourth week, with 32.45 and 62.54% for the CBE and L-CBE creams, respectively (Figure 16, Figure 17, Figure 18 and Figure 19).

There was no significant difference in skin elasticity (R2, R5, and R7) between the L-CBE and CBE groups 2 weeks following the operation. However, the L-CBE group had a significantly higher level of elasticity (R2, R5, and R7) than the CBE group after using the creams for 4 weeks. These findings may be due to the CBE’s increase in skin collagen, which is predominantly connective tissue that is found in the dermis and plays a major role in providing a flexible and taut skin layer [66]. Collagen alterations are fundamental to the skin aging process, in that as people age, the levels of collagen decline, leading to wrinkles [67]. The results correspond to the report of an in vivo test in which Girsang applied 10% coffee bean extract cream to the skin of rat tails that had been shaved for 4 weeks, boosting the collagen level by 91.30% [18]. Rodrigues also founded that the application of coffee silverskin extract exhibited equivalent efficiency to hyaluronic acid in reducing deep wrinkles in the crow’s feet area over the course of 28 days [68]. In addition, 0.5% of the caffeine in a gel base considerably enhanced forehead skin elasticity after 4 weeks of use [69]. Furthermore, the L-CBE cream appeared to significantly increase skin elasticity to a greater degree than the CBE cream (*p* < 0.05) by the end of the experiment. This might be a result of the liposome system enabling a higher level of active chemical penetration in the skin. The results indicate that CBE has the potential to be employed for the purpose of boosting skin elasticity and slowing down the signs of skin aging. It can also be loaded into liposome formulations to increase its effectiveness in skin anti-aging cosmetic and cosmeceutical products.

Human skin color is influenced by the pigment melanin, which is found in the skin. Therefore, melanin levels can be distinguished between darker and lighter skin. In this study, Mexameter was used to detect the amount of melanin in the epidermis beneath the skin’s surface in order to assess the brightening effects of the CBE and the L-CBE creams on volunteer subjects’ skin at weeks 0, 2, and 4.

**Figure 20:**
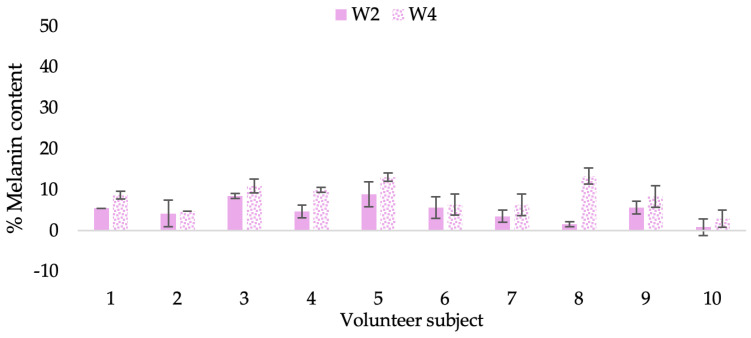
Changing percentage of melanin content of each volunteer subject after application of B cream.

**Figure 21:**
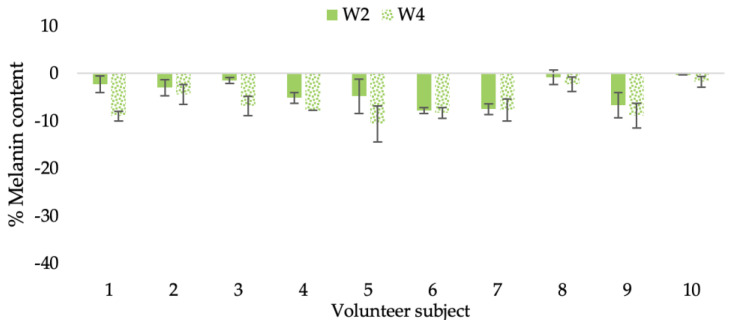
Changing percentage of melanin content of each volunteer subject after application of CBE cream.

**Figure 22:**
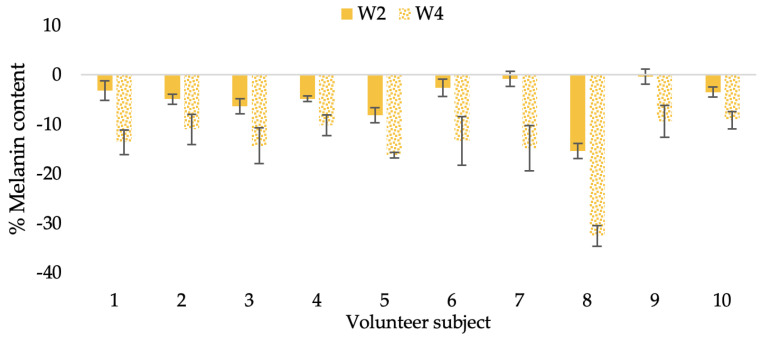
Changing percentage of melanin content of each volunteer subject after application of L-CBE cream.

**Figure 23:**
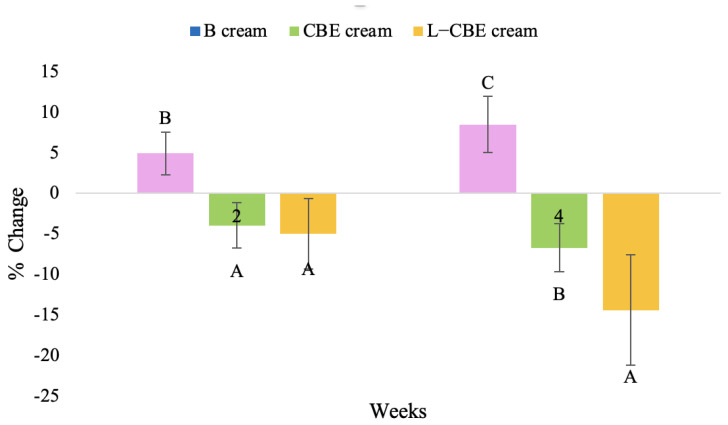
Changing percentage of melanin content of all volunteer subjects after product application. Data with different letters (A, B, and C) indicate significant differences (*p* < 0.05) among creams.

The findings demonstrated that volunteer subjects who used the CBE and the L-CBE creams tended to have lower melanin contents than the baseline (Figure 20, Figure 21, Figure 22 and Figure 23). After 2 weeks, it was evident that the CBE and the L-CBE creams could lower melanin levels by 3.97 and 4.99% compared to the initial value. At week 4, the melanin content had dramatically decreased from the beginning, with 6.71 and 14.43% for the CBE and the L-CBE creams, respectively (*p* < 0.05). In comparison to the placebo cream, both creams significantly outperformed it in terms of reducing melanin. This was due to the presence of numerous significant phenolic and alkaloid chemicals in coffee extract, which strongly inhibit the tyrosinase enzyme and melanin synthesis [70,71,72,73,74,75]. In addition, the liposome cream showed a significantly higher efficacy of melanin reduction than the CBE cream (*p* < 0.05) at week 4. The findings suggest that the CBE can be used to lighten skin and can be included into the liposome encapsulation system, increasing its effectiveness.

## 3. Materials and Methods

### 3.1. Reagents and Chemicals

Phosphatidylcholine, cholesterol, caffeine, chlorogenic acid, Folin–Ciocalteu reagent, gallic acid, lipopolysaccharide (LPS), phenylmethanesulfonyl fluoride, sodium carbonate, Tris/HCl, triton x-100, vanillin, and SOD assay Kit-WST were purchased from Sigma-Aldrich Co., St. Louis, MO, USA. Ethanol, sulfuric acid, and trichloroacetic acid were purchased from Merck, Darmstadt, Germany. Ferric chloride and potassium ferricyanide were purchased from Fisher Scientific, Waltham, MA, USA. Dimethyl sulfoxide (DMSO) and 3-(4,5-dimethylthiazol-2-yl)-2,5-diphenyltetrazolium bromide (MTT) were purchased from Bio Basic Inc., Markham, ON, Canada. Fetal bovine serum, fibroblast, penicillin streptomycin solution, phosphate-buffered saline (PBS), and typsin/EDTA solution were purchased from Gibco, Grand Island, NY, USA. The CBE was obtained as a gift from The NIC Company Limited, Chiang Rai, Thailand.

### 3.2. Identification of Chlorogenic Acid and Caffeine Using HPLC

The major chemical constituents of the CBE, chlorogenic acid and caffeine, were determined using high-performance liquid chromatography (HPLC, 1290 Infinity II LC System, Agilent Technologies, Waldbronn, Germany) with photodiode array (PDA) detection at 280 nm [45]. After being filtered via Whatman^®^ (Maidstone, UK) membrane filters with nylon pore sizes of 0.2 μm, the CBE was then injected into a Poroshell 120 EC-C18 (4.6 × 250 mm, 4 μm) with 10 μL of the extract. The mobile phase systems included (A) acetonitrile and 1.5% acetic acid in a 10:90 ratio and (B) acetonitrile and 1.5% acetic acid in a 15:85 ratio. Mobile phase A was eluted from 0 to 6 min, while mobile phase B was eluted from 6 to 12 min, both at flow rates of 1 mL/min. The standard retention times for chlorogenic acid and caffeine were 6.139 and 6.441 min, respectively. The chromatograms were plotted and processed using Agilent software (version C.01.07 [22]) and the peak areas were automatically integrated. Peak areas acquired from HPLC analysis were plotted against standard solution concentrations to construct the standard calibration curves. The stock solutions of caffeic acid and chlorogenic acid were dissolved in DMSO and diluted to obtain concentrations of 5, 10, 25, 50, and 100 μg/mL for the calibration range. Linear regression was used to fit the calibration curves of both standards. Using the calibration curve of the standards, the concentrations of chlorogenic acid and caffeine were estimated.

### 3.3. Preparation and Characterization of Nanoliposomes

#### 3.3.1. Nanoliposome Preparation

In this study, the liposomes were prepared by the film method. Briefly, 15 g of phosphatidylcholine (PC) and 1 g of cholesterol (CL) were dissolved in 60 mL of ethanol with constant stirring at 50 °C for 30 min. Then, the solvent was removed using an evaporator (Buchi R-114 Rotary Vap System, BUCHI Corporation, New Castle, DE, USA) at 40 °C until it formed a thin film, which was further mixed with 30 mL of deionized water using a homogenizer (T25 digital ultra turrax, IKA, Staufen, Germany) at 8000 rpm for 30 min. After an opaque solution was obtained, the CBE (5%) was added and mixed using a homogenizer at 5000 rpm for 10 min. Then, the liposomes’ particle sizes were decreased by feeding them through a high-pressure microfluidizer (LM20 Microfluidizer, Microfluidics, Westwood, MA, USA) at 25,000 psi for 5 cycles.

#### 3.3.2. Particle Size and Zeta Potential Measurement

The sample particle size, polydispersity index (PDI), and zeta potential were measured by Zetasizer (Zetasizer Nano S, Malvern Instruments, Worcestershire, MA, USA). The sample (0.01%, *w*/*v*) was suspended in water and sonicated with an ultrasonic bath (DT 255 H, Bandelin Co., Berlin, Germany) for 5 min at 20 KHz for complete dispersion of particles.

#### 3.3.3. Encapsulation Efficiency and Loading Efficiency

The encapsulation efficiency (EE) based on total phenolic content was determined using spectrophotometry. Encapsulation efficiency quantifies the percentage of successfully encapsulated active substances. Briefly, liposomes (10 g) were centrifuged at 9000 rpm at 4 °C for 30 min. The supernatant and precipitate were separately collected. The precipitate was redissolved in ethanol. Both solutions were measured for their total phenolic content using the Folin–Ciocalteu method, and chlorogenic acid was used as a standard compound. Finally, the EE and LE were expressed as percentages through the following equation.

(1) $$\%\;EE=\frac{APC-SPC}{APC}\times 100$$

where SPC is total phenolic content of the supernatant (surface total phenolic content) and APC is the added total phenolic content in the liposome preparation process.

(2) $$\%\;LE=\frac{IPC}{WL}\times 100$$

where IPC is total phenolic content of the precipitate (inner total phenolic content) and WL is the weight of L-CBE.

#### 3.3.4. Stability Test

In order to determine the effect of accelerated conditions on the stability of the CBE and the L-CBE, the method was performed following the protocol of Marx [76]. Briefly, 50 g of samples were weighed in a wide-mouth glass jar with closed-screw cap; then, the prepared samples were stored in various conditions, including at a low temperature (4 °C) in a refrigerator, at room temperature (RT), and at a high temperature (45 °C) in a hot air oven, for 12 weeks. Before and during testing, samples were analyzed and recorded as the initial and remaining amounts of total phenolic contents, which used the standard calibration curve of chlorogenic acid. All samples were investigated in triplicate at baseline and at time durations of 2, 4, 8, and 12 weeks.

#### 3.3.5. In Vitro Permeation Measurement

The permeation experiment was studied according to the method described by Mattiasson [77] using a Franz diffusion cell. Cellulose acetate membranes were washed prior to use to remove any dirt that could interfere with the execution of the experiments or impregnation. Washing was performed by soaking the membranes in phosphate-buffered saline (PBS), pH 7.4, for 60 min. The membrane was mounted on a receiver cell with the stratum corneum side facing the donor cell. During the experiment, the receiver cells were filled with PBS (pH 7.4), which was continuously stirred at 300 rpm. The temperature (37 ± 0.2 °C) was maintained with an external circulating water bath. In the donor cell, 1 mL samples of 1 mg/mL were separately applied homogeneously on the membrane. At pre-set time points (0.5, 1, 1.5, 3, 5, 7, 24, 48, and 100 h), 1 mL of the receptor solution was withdrawn, and the same volume of fresh receptor was added to the receptor cell in order to maintain the condition. Samples were measured for total phenolic content using the Folin–Ciocalteu method.

### 3.4. Cytotoxicity

The cytotoxicity assessment was performed by MTT assay [78]. Human dermal fibroblast (HDF) cells (100 µL), suspended in a complete culture medium at a density of 1 × 10^6^ cells/mL, were placed into a 96-well plate for 24 h. Then, cells were treated with 100 µL of various sample concentrations (0–20 mg/mL) in media for 24 h. Afterward, the culture medium was removed. Then, 0.5 mg/mL of MTT solution (100 µL) was added to each well and incubated for 4 h. DMSO (100 µL) was added to all wells and incubated for 30 min at room temperature. The absorbance of each well was measured at 570 nm using a microplate reader (UVM 340, Biochrom, Holliston, MA, USA). The percentage of cell viability was calculated by the following formula, and the IC_50_ was expressed.

(3) $$Viable\;cell\left(\%\right)=\frac{A_{treated\;group}}{A_{untreated\;group}}\times 100$$

### 3.5. Anti-Aging Activity

#### 3.5.1. Superoxide Dismutase Activity

Fibroblast cells were supplemented with 100 μL of samples and incubated in a 5% CO_2_ humidified incubator at 37 °C for 24 h. Then, cells were harvested with 0.05% typsin solution. Cells were lysed with lysis buffer and centrifuged at 3000 rpm for 15 min (Vichit and Saewan, 2016). The SOD activity in the supernatants was measured using a SOD assay kit (Sigma-Aldrich, USA). A microplate reader was used to measure the absorbance at 450 nm after the mixture of the supernatant (20 µL), WST working solution (200 µL), and enzyme working solution (20 µL) was incubated at 37 °C for 20 min (Biochrom, Holliston, MA, USA). The following equation was used to compute the SOD activity:

(4) $$\%\;SOD\;activity=\frac{A_{control}-A_{sample}}{A_{control}}\times 100$$

#### 3.5.2. Nitric Oxide Inhibition

Fibroblast cells (20,000 cells/mL, 100 uL) were supplemented with 100 μL samples before being stimulated with 1 μg/mL lipopolysaccharide (LPS) and incubated for 24 h [79]. A 50 μL solution of sulfanilamide in 5% phosphoric acid was added to the culture medium (50 μL). After adding 50 μL of 0.1% N-1-napthylethylenediamine dihydrochloride aqueous solution, the absorbance at 540 nm was measured. Using a sodium nitrite standard curve, the quantity of nitrite in the sample was measured. The following equation was used to calculate the nitric oxide inhibition percentage:

(5) $$\%\;inhibition\;of\;nitric\;oxide=\frac{A_{control}-A_{sample}}{A_{control}}\times 100$$

#### 3.5.3. Collagenase Inhibition

The collagenase inhibitory activity was assessed using a MMP-1 colorimetric drug discovery kit, which was created to screen MMP-1 inhibitors utilizing a thiopeptide as a chromogenic substrate. Human dermal fibroblast cells (20,000 cells/mL, uL) were supplemented with a 100 µL sample and incubated for 24 h. Cells were then harvested using a 0.05% trypsin solution. After being lysed with a lysis buffer, the cells were centrifuged at 3000 rpm for 15 min. The supernatants, 20 µL of 153 mU/µL MMP-1 and 20 μL of 1.3 µM prototypic control inhibitor, were mixed and incubated at 37 °C for 60 min to allow for interaction between the inhibitor and the enzyme. Following the addition of 10 μL of 100 µM thiopeptide, the absorbance was measured at 412 nm. This formula was used to determine the collagenase inhibition percentage:

(6) $$\%\;Collagenase\;inhibition=\frac{A_{control}-A_{sample}}{A_{control}}\times 100$$

### 3.6. Formulation

The CBE and L-CBE creams were prepared according to the O/W emulsion base cream, as displayed in Table 1. In parts A and B, all ingredients were separately weighed and heated to 70 °C. Then, part A was added into part B with constant stirring until well mixed. Part C was added into part AB and mixed using a homogenizer (T25 digital ultra turrax, IKA, Staufen, Germany) at 6000 rpm for 15 min. After that, the mixture was cooled down to 40 °C, and part D was added into part ABC, then homogenized using a homogenizer. The free CBE and L-CBE, at concentrations of 5%, were separately incorporated into the prepared base cream, and CBE and L-CBE creams were obtained. Phase separation did not appear in either cream when centrifuged at 9000 rpm for 30 min. The products were evaluated for characteristics such as appearance, color, texture, and spreadability by visual observation and skin application. The products were measured for their pH values using a pH meter (HI2211 HANNA Basic pH/ORP Benchtop Meter, Hanna Instruments, Bangkok, Thailand).

### 3.7. Clinical Study

#### 3.7.1. Ethical Aspects

The protocol was approved by the Mae Fah Luang University Ethics Committee under approval number EC21228-17. The research protocols were followed in accordance with the Helsinki Declaration on human subjects. Before taking part in the clinical study, each participant signed a written informed agreement that detailed the nature of the study, the steps to be taken, the general characteristics of the substances being evaluated, and any known or predicted negative effects that could arise from participation.

#### 3.7.2. Subjects

The 30 participants in this study were concerned about their skin’s tone and dullness, wrinkles, and fine lines. Men and women with normal skin and Fitzpatrick skin types II-IV, aged 20 to 60 years, met the primary inclusion criteria for this study. The routine usage of other skincare products at the same time as the study’s duration was prohibited. Subjects were selected excluding those who did not meet the clinical criteria due to factors that could interfere with the test’s results. Those who had a chronic skin condition, sensitive or easily irritated skin, were pregnant, who regularly smoked or drank alcohol, and those who had taken part in the same experiment within the previous month were excluded from the study.

#### 3.7.3. Skin Irritation Testing

Skin irritation testing was carried out utilizing a Draize model [80]. A single occlusive patch test was performed on the subjects. The upper arm of the volunteer subjects was covered with Finn chambers^®^ at a size of 8 mm (Smartpractice, McDowell, WV, USA) which were used to observe skin irritation for 24 h. Each chamber was saturated with B cream as a placebo, CBE cream, L-CBE cream, 0.5% *w*/*v* of sodium lauryl sulfate as a positive reaction, and deionized water as a negative reaction. Then, at 30 min and at 24 h after removing the patch, erythema and edema were observed. The mean irritation index (M.I.I.) was utilized to evaluate each test material (Table 2 and Table 3).

#### 3.7.4. Efficacy Test

Everyone who passed the occlusive single patch test without experiencing any irritation (M.I.I score 0.5) was eligible to take part in this study. To prevent bias, a number rather than a name was given to each volunteer. Three treatment groups were randomly assigned to the volunteer subjects. Group 1 received the B cream. The CBE cream was given to group 2, while the L-CBE cream was given to group 3. Volunteer subjects, who received instruction on how to use the cream, applied 0.5 g of the tested creams onto their faces twice daily at home in the morning and before bedtime under normal conditions for 4 weeks. In particular, the study’s participants were instructed not to use any other skincare products and to avoid sunlight as much as possible. Before measurements were taken, each volunteer’s face was cleansed with a gentle facial cleanser, and individuals spent at least 30 min acclimating to the room’s conditions. All skin measurements were performed in a controlled environment at a constant temperature (20 ± 5 °C) and relative humidity (60 ± 5%). By using the single-blind method, measurements of the skin’s elasticity and melanin content were taken in triplicate at week 0 (baseline), week 2, and week 4 following application. Facial skin elasticity was measured using Cutometer^®^ dual MPA 580 (Courage Khaazaka electronic GmbH, Cologne, Germany) by assessing the net elasticity of the skin without viscous deformation, and this was repeated three times to obtain an accurate measurement of the same part by pressing the measurement probe onto the skin. The melanin content of the skin was measured using a Mexameter (MX18, Courage Khaazaka electronic, Cologne, Germany). The content of melanin was expressed as the mean value, which was calculated and compared following sensor contact at the selected site of the skin’s surface.

### 3.8. Statistical Analysis

IBM SPSS statistics software version 23 (SPSS Inc., Chicago, IL, USA) was used to conduct the statistical analysis. The mean and standard deviation were used to express continuous variables (SD). The data were presented as the triplicate values’ means and standard deviations. Each experiment’s statistical comparisons were carried out using a paired *t*-test with a 95% confidence level. Statistical significance was defined as a *p*-value of 0.05.

## 4. Conclusions

In this study, the major compound in CBE was found to be chlorogenic acid (131.85 µg/mL) by HPLC analysis. The CBE was successfully loaded in nanosized liposomes with a high EE of 71.26%. The liposomal encapsulation system enhanced the stability of chlorogenic acid, especially when stored under high thermal conditions. It provided a higher controlled release and penetration of active compounds through an artificial membrane than the free extract did. The liposomes showed lower cytotoxicity than the free extract, while its anti-aging activities, NO inhibition and SOD activity, and anti-collagenase inhibition in HDF cells were slightly higher than those of the free extract. The clinical evaluation demonstrated that the L-CBE cream significantly enhances skin elasticity and decreases melanin content more effectively than the free extract. To our knowledge, this present work is the first report of the clinical efficacy of CBE encapsulation in nanoliposomes. The results indicate that a satisfactory encapsulation efficacy of CBE in a lipid-nanocarrier, which could enhance stability, lower toxicity, and maintain biological activity, has the potential to be used as an active ingredient for anti-aging and skin brightening in cosmetic and cosmeceutical products.

## Figures and Tables

**Table 1:** The formulation of the CBE and L-CBE creams.

Part	Ingredients	% *w*/*w*	Function
A	Water	q.s. to 100	Solvent
	Butylene Glycol	2.0	Humectant
	Glycerin	5.0	Humectant
B	Glyceryl Stearate SE	1.2	Emulsifier
	Cetearyl Alcohol	1.5	Emollient
	Glyceryl Stearate SE (and) PEG-100 Stearate	1.5	Emulsifier
C	Acrylates/Acrylamide Copolymer (and) Mineral Oil (and) Polysorbate 85	1.5	Thickener/Emulsifier
D	Phenoxyethanol	0.8	Preservative
	CBE or L-CBE	5.0	Active

**Table 2:** Scores for erythema, edema, and other skin irritations.

Scores	Clinical Description	
	Erythema	Edema
0	No erythema	No edema
1	Light erythema (hardly visible)	Light edema (hardly visible)
2	Clearly visible erythema	Light edema
3	Moderate erythema	Moderate edema (about 1 mm raised skin)
4	Serious erythema (dark red with possible formation of light scars)	Strong edema (extended swelling even beyond the application area)

**Table 3:** Classification of M.I.I. (according to the amended Draize classification).

M.I.I	Classification
<0.50	Non-irritation
From 0.50 to <2.00	Slightly irritation
From 2.00 to <5.00	Moderately irritation
From 5.00 to <8.00	Strongly irritation

## Data Availability

All the data are available within the manuscript.

## Sample Availability

Samples of CBE and liposomal CBE are available from the authors.
